# DCL‐suppressed *Nicotiana benthamiana* plants: valuable tools in research and biotechnology

**DOI:** 10.1111/mpp.12761

**Published:** 2018-12-19

**Authors:** Konstantina Katsarou, Eleni Mitta, Eirini Bardani, Anastasis Oulas, Elena Dadami, Kriton Kalantidis

**Affiliations:** ^1^ Institute of Molecular Biology and Biotechnology Foundation for Research and Technology‐Hellas Heraklion Greece; ^2^ Department of Biology University of Crete Heraklion Greece; ^3^ Present address: Bioinformatics Group, The Cyprus Institute of Neurology and Genetics Nicosia Cyprus; ^4^ Present address: RLP AgroScience, AlPlanta Neustadt Germany

**Keywords:** CMV, DCL, Dicer, GFP, miRNA, *Nicotiana benthamiana*, RNAi, TRV, TuMV

## Abstract

RNA silencing is a universal mechanism involved in development, epigenetic modifications and responses to biotic and abiotic stresses. The major components of this mechanism are Dicer‐like (DCL), Argonaute (AGO) and RNA‐dependent RNA polymerase (RDR) proteins. Understanding the role of each component is of great scientific and agronomic importance. Plants, including *Nicotiana benthamiana*, an important plant model, usually possess four DCL proteins, each of which has a specific role, namely being responsible for the production of an exclusive small RNA population. Here, we used RNA interference (RNAi) technology to target DCL proteins and produced single and combinatorial mutants for DCL. We analysed the phenotype for each DCL knockdown plant, together with the small RNA profile, by next‐generation sequencing (NGS). We also investigated transgene expression, as well as viral infections, and were able to show that DCL suppression results in distinct developmental defects, changes in small RNA populations, increases in transgene expression and, finally, higher susceptibility in certain RNA viruses. Therefore, these plants are excellent tools for the following: (i) to study the role of DCL enzymes; (ii) to overexpress proteins of interest; and (iii) to understand the complex relationship between the plant silencing mechanism and biotic or abiotic stresses.

## Introduction

Plants have developed sophisticated pathways to respond to environmental stimuli. One such mechanism is RNA silencing, which is capable of regulating gene expression in response to various biotic and abiotic stresses (Khraiwesh *et al*., 2012; Pumplin and Voinnet, 2013). Key proteins involved in this complex mechanism are ribonucleases named Dicer‐like proteins (DCL) (Martínez de Alba *et al.*, 2013), with most plants, including *Nicotiana benthamiana*, encoding four distinct DCL proteins (Nakasugi *et al.*, 2013).

Each DCL carries out a specific function. DCL1 is involved in the micro‐RNA (miRNA) biogenesis pathway producing 21‐nucleotide (21‐nt) small RNAs from miRNA precursors which are transcribed by a complex of proteins, including RNA polymerase II. Primary miRNAs are initially trimmed by DCL1 to precursor miRNAs from which the protein further excises the miRNA/miRNA* duplexes (Achkar *et al.*, 2016). After excision, miRNAs are bound to a protein complex, involving at least one Argonaute (AGO) protein (RNA‐induced silencing complex, RISC), and are directed to complementary RNA sequences to be silenced (Martínez de Alba *et al.*, 2013). Additional roles of DCL1 have also been described in DNA methylation and transposon silencing (Laubinger *et al.*, 2010). DCL1 is preferentially transcribed from the maternally inherited allele and is essential for the development of the embryo; thus, homozygous mutations in this gene are embryo lethal (Bozorov *et al.*, 2012; Castle *et al.*, 1993; Ray *et al.*, 1996).

The DCL2 protein processes double‐stranded RNA (dsRNA) molecules of exogenous origin and natural antisense small interfering RNAs (siRNAs) into 22‐nt primary siRNAs (Borsani *et al.*, 2005; Xie *et al.*, 2004). DCL2 is mainly involved in antiviral defence; however, there is mounting evidence that its role is masked by DCL4 activity, as it has been clearly demonstrated that DCL2 is also involved in transitivity and the RNA decoy mechanism (Mlotshwa *et al.*, 2008; Parent *et al.*, 2015; Qin *et al.*, 2017; Taochy *et al.*, 2017; Zhang *et al.*, 2015).

DCL3 is involved in the production of 24‐nt small RNAs which operate in the RNA‐dependent DNA methylation (RdDM) pathway (Blevins *et al.*, 2015; Qi *et al.*, 2005; Xie *et al.*, 2004; Xie and Yu, 2015). DCL3 has also been reported to produce long miRNAs of 24 nt in *Arabidopsis thaliana*, rice, tomato and *N. benthamiana* (Kangquan *et al.*, 2015; Kravchik *et al.*, 2014a; Vazquez *et al.*, 2008; Wu *et al.*, 2010), and also phased siRNAs in rice (Song *et al.*, 2012).

DCL4 is the primary DCL enzyme involved in antiviral defence, as DCL4 *A. thaliana* mutants are more susceptible to different viruses (Bouché *et al.*, 2006; Deleris *et al.*, 2006; Henderson *et al.*, 2006). DCL4 is responsible for the production of 21‐nt‐long siRNAs and is further involved in multiple endogenous processes, such as the production of 21‐nt trans‐acting siRNAs (tasiRNAs), which regulate major developmental processes (D'Ario *et al.*, 2017; Pulido and Laufs, 2010), the production of miR822, miR839 and miR869 in *A. thaliana *(Ben Amor *et al.*, 2009; Rajagopalan *et al.*, 2006) and, finally, in transcription termination (Duc *et al.*, 2013; Liu *et al.*, 2012). Although the role of each DCL protein is, to some extent distinct. It is to note that important functional redundancies have also been reported, especially in *A. thaliana* (Blevins *et al.*, 2006; Bouché *et al.*, 2006; Deleris *et al.*, 2006; Gasciolli *et al.*, 2005; Henderson *et al.*, 2006; Kasschau *et al.*, 2007; Xie *et al.*, 2005).

At present, the majority of studies addressing the role of DCL proteins have been performed with *A. thaliana* knockout plants. This approach has proven to be very fruitful indeed, as it has shed light onto key aspects of the RNA silencing pathways. Nevertheless, there are certain limitations to this methodology. (i) Although different mutants of the same DCL are often treated as equally informative, the plants used are usually either T‐DNA or point mutants with different phenotypes, depending on the efficiency of the mutation. To the point at hand, the analysis of three *A. thaliana dcl4* mutants (*dcl4‐6*, *dcl4‐70b1* and *dcl4‐8*) showed differences in the processing of mir822, TAS3 and TAS1, respectively (Montavon *et al.*, 2018). (ii) *Arabidopsis thaliana* is a major plant model, but not necessarily the most representative species to address all plant phenomena. *Arabidopsis thaliana *cannot be infected by certain viruses and viroids, is not ideal for the study of systemic processes and is not used for fast transient methods, such as agroinfiltration or virus‐induced gene silencing (VIGS) experiments (Daròs and Flores, 2004; Goodin *et al.*, 2008; Wroblewski *et al.*, 2005).

In order to overcome these limitations and to address the function of DCL proteins in other plant models, we aimed to generate *N. benthamiana* DCLi knockdown plants using RNA interference (RNAi). *Nicotiana benthamiana* is a plant species of the Solanaceae family. *Nicotiana benthamiana* is susceptible to many plant viruses, allows for relatively easy experiments on intercellular macromolecular movement, as well as VIGS experiments, and its genome has been sequenced recently and made publically available (Bally *et al.*, 2015; Bombarely *et al.*, 2012; Goodin *et al.*, 2008; Nakasugi *et al.*, 2013). In this study, we present the molecular and phenotypic characterization of plants suppressed for each and every DCL, as well as their combinations. We show that these plants can be used in multiple experimental set‐ups, such as for the study of various aspects of DCL functions, transient expression, viral infection studies, as well as their interplay with RNAi pathways.

## Results and Discussion

The alteration of protein expression in living organisms has been a scientific goal for many years. Two methods commonly used to suppress the expression of a specific protein are genome editing [mainly CRISPR/Cas9 (Clustered Regularly Interspaced Short Palindromic Repeats/CRISPR‐associated 9) technology] and RNAi. CRISPR/Cas9 has been developed in the last few years and has been successfully used for different research purposes in different systems (Bai *et al.*, 2018; Bier *et al.*, 2018; Kell *et al.*, 2018). CRISPR/Cas9 has a number of advantages, including the generation of complete knockouts without necessarily transgenic sequences present in the final mutant. Nevertheless, this technique is rather laborious, especially when multiple alleles are to be targeted, may lead to off‐target effects and usually has a requirement for homozygosity (Khan Ullah *et al.*, 2017). In addition, lethality may occur if the targeted gene is essential in specific developmental stages. As a result, there is still a need for RNAi as a powerful, reliable and fast method to knock down single or multiple gene(s).

In 2013, we generated RNAi‐suppressed DCL lines using hairpins against each and every identified DCL protein (Dadami *et al.*, 2013). At least two plant lines were obtained per DCL (DCL1.9i, DCL1.13i, DCL2.11i, DCL2.41i, DCL3.1i, DCL3.10i, DCL4.9i and DCL4.16i) to account for possible T‐DNA insertion bias. We also created two lines producing a hairpin for both DCL2 and DCL4 (DCL2/4.5i and DCL2/4.16i) (Dadami *et al.*, 2013). DCLi plant lines were crossed with each other to produce plants with combined DCL suppression. Effective down‐regulation of the targeted DCL mRNAs in all of these lines has been validated previously (Dadami *et al.*, 2013; Katsarou *et al.*, 2016), but is also summarized in Table S1 (see Supporting Information) for a clearer understanding of the data presented here.

In all generated plant lines, with the exception of DCL1, the introduced hairpin was able to efficiently suppress the targeted DCL, even in double‐ or triple‐crossed plants. Especially in the case of DCL3.10(x)2/4.5i plants, all three DCLs were found to be strongly suppressed, showing that AGO complexes can efficiently achieve RNAi with a minimal supply of small RNAs produced, confirming the high effectiveness of the RNAi machinery. These plants have already been successfully used for research purposes, proving their importance for the scientific community (Cordero *et al.*, 2017; Dadami *et al.*, 2013; Katsarou *et al.*, 2016). With this study, we intended to broaden the characterization of DCLi plant lines for further scientific or other uses.

### Phenotypic analysis of DCLi plants

**Figure 1:**
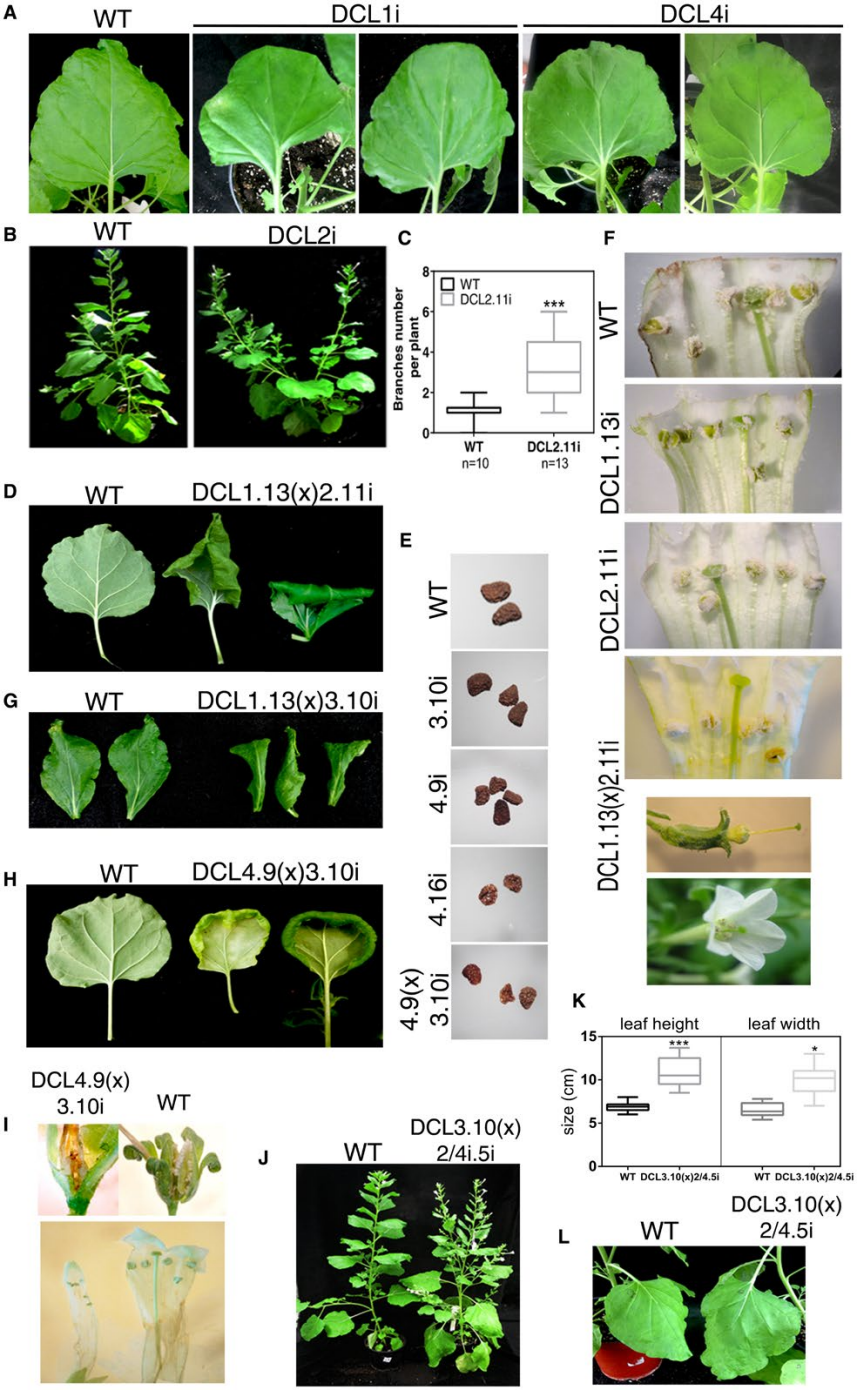
Phenotypic analysis of DCLi and DCLi crossed plants. (A, D, G, H) Leaves of wild‐type (WT), DCL1i, DCL4i, DCL1.13(x)2.11i, DCL1.13(x)3.10i and DCL4.9(x)3.10i plant lines. (B, J, L) Photographs of WT, DCL2i, DCL3.10(x)2/4.5i plant lines. (E) Photographs of seeds from WT, DCL3.10i, DCL4.9i, DCL4.16i and DCL4.9(x)3.10i plant lines. (F, I) Flowers of WT, DCL1.13i, DCL2.11i, DCL1.13(x)2.11i and DCL4.9(x)3.10i plant lines. (C, K) Graph representing the branch numbers of DCL2i and leaf height/width (cm) measurements of DCL3.10(x)2/4.5i plants (leaf number 8 in plants at the stage of 12 leaves). Unpaired Student’s *t*‐test was performed with ***P < 0.001 and *P < 0.05. [Colour figure can be viewed at wileyonlinelibrary.com]

First, we focused on the phenotypic characterization of the DCLi plants. To our knowledge, this is the first time that phenotypic analysis for DCL suppression has been made in *N. benthamiana*. In our growth conditions, single *N. benthamiana* DCLi plants presented minor phenotypic differences, as shown in Fig. 1A. NbDCL1.9i and NbDCL1.13i showed leaf distortion in a stochastic manner. As there is no homogeneity (especially in *Arabidopsis*) in *dcl* knockout plants, it is difficult to correlate these phenotypes with the observations in previous studies. *Arabidopsis thaliana* DCL1i plants showed significant phenotypic variability (Lackey *et al.*, 2010), which correlates with the phenotypic stochasticity observed here, whereas *Oryza sativa* DCL1i plants showed severe dwarfism, curly leaves and tortuous shoots (Liu *et al.*, 2005). *Physcomitrella patens dcl1a* null mutants presented abnormality in cell size, shape and growth rate (Khraiwesh *et al.*, 2010) and, finally, trans‐activated DCL1i plants showed malformation in leaf primordia (Kravchik *et al.*, 2014b). NbDCL2i plants showed increased branching (Fig. 1B,C), which was not the case for loss‐of‐function *dcl2 A. thaliana* mutants or knockout *Nicotiana attenuata *plants (Bozorov *et al.*, 2012; Gasciolli *et al.*, 2005). NbDCL3i plants did not show any evidence of a phenotypic malformation at least until generation T10, unlike results in other plant models (Bozorov *et al.*, 2012; Gasciolli *et al.*, 2005; Kravchik *et al.*, 2014a)*. *Finally, NbDCL4i plants presented rounded leaves and decreased seed production, combined with significant malformations, especially in the DCL4.16i line (Fig. 1A,E). Furthermore, scanning electron microscopy analysis of the leaf surface revealed a decrease in trichome and stomata number, combined with an increase in stomatal size (Fig. S1, see Supporting Information). These results partially correlate with the observed phenotype for *Arabidopsis*, tomato and rice plants, for which distorted leaves, altered trichome production and male sterility have been described (Bouché *et al.*, 2006; Gasciolli *et al.*, 2005; Song *et al.*, 2012; Xie *et al.*, 2005; Yifhar *et al.*, 2012).

The majority of the crossed plant lines showed pronounced developmental malformations, the latest at the F3 generation. More specifically, DCL1.13i(x)DCL2.11i showed stunted growth compared with wild‐type (WT), DCL1.13i or DCL2.11i plants, and leaves curled downwards forming a triangle (Fig. 1D). However, the abaxial and adaxial surfaces seemed to grow at a similar rate, as the leaf could be stretched and opened manually. NbDCL1.13i(x)DCL2.11i flowers had their pistil extended outside the corolla early on during development, which was not observed in either DCL1.13i or DCL2.11i (Fig. 1F). Nevertheless, male and female fertility did not seem to be affected. Furthermore, there was increased trichome and stomata number per area compared with WT plants (Fig. S1). These phenotypes are in contrast with what has been reported in *Arabidopsis*, where no differences in the severity of the phenotype were observed between *dcl1* and *dcl1‐9/dcl2‐4* plants (Bouché *et al.*, 2006; Gasciolli *et al.*, 2005).

Compared with WT plants, in NbDCL2.11i(x)3.10i, no phenotypic alterations were observed, even after three generations, contradicting that which has been proposed for *A. thaliana dcl2xdcl3* plants. In this case, stronger phenotypic defects—which increase with generations—have been described, possibly because of the accumulation of epigenetic alterations (Gasciolli *et al.*, 2005). Either this discrepancy is a result of differences in the crosstalk of DCL2 and DCL3 pathways in these two species, or it is plausible that the remaining amount of DCL protein is sufficient to maintain normal function of the epigenetic pathway. Ten‐week‐old DCL1.13i(x)3.10i plants presented a twist of the upper leaves compared with WT plants (Fig. 1G). Heart‐shaped leaves, flower malformation, enlarged stomata and seed defects were observed in DCL4.9(x)3.10i plants, showing a more pronounced phenotype compared with individual DCL3i or DCL4i plants, in accordance to that which has been reported for *A. thaliana* and *N. attenuata *(Figs 1H,I and S1) (Bozorov *et al.*, 2012; Gasciolli *et al.*, 2005). Finally, NbDCL3.10(x)2/4.5i plants showed larger leaves than the WT, combined with increased branching (Fig. 1J–L). This was also the case with the *Arabidopsis* triple mutant (*dcl2*‐4/*dcl3*‐1/*dcl4*‐1) plants used in the work of Bouché *et al.* (2006), but not of Henderson *et al.* (2006), where the use of a different mutant (*dcl2*‐1/*dcl3*‐1/*dcl4*‐2) produced plants with strong developmental defects.

Taken together, these results suggest that each DCL protein is involved in different aspects of plant development as each single line presented its own phenotypic alterations. In addition, double or triple RNAi mutants demonstrated pronounced phenotypic abnormalities compared with single plant lines, further supporting a functional overlap of DCL proteins in multiple developmental pathways.

### Profiling and analysis of small RNAs in *N. benthamiana* DCLi mutants

**Figure 2:**
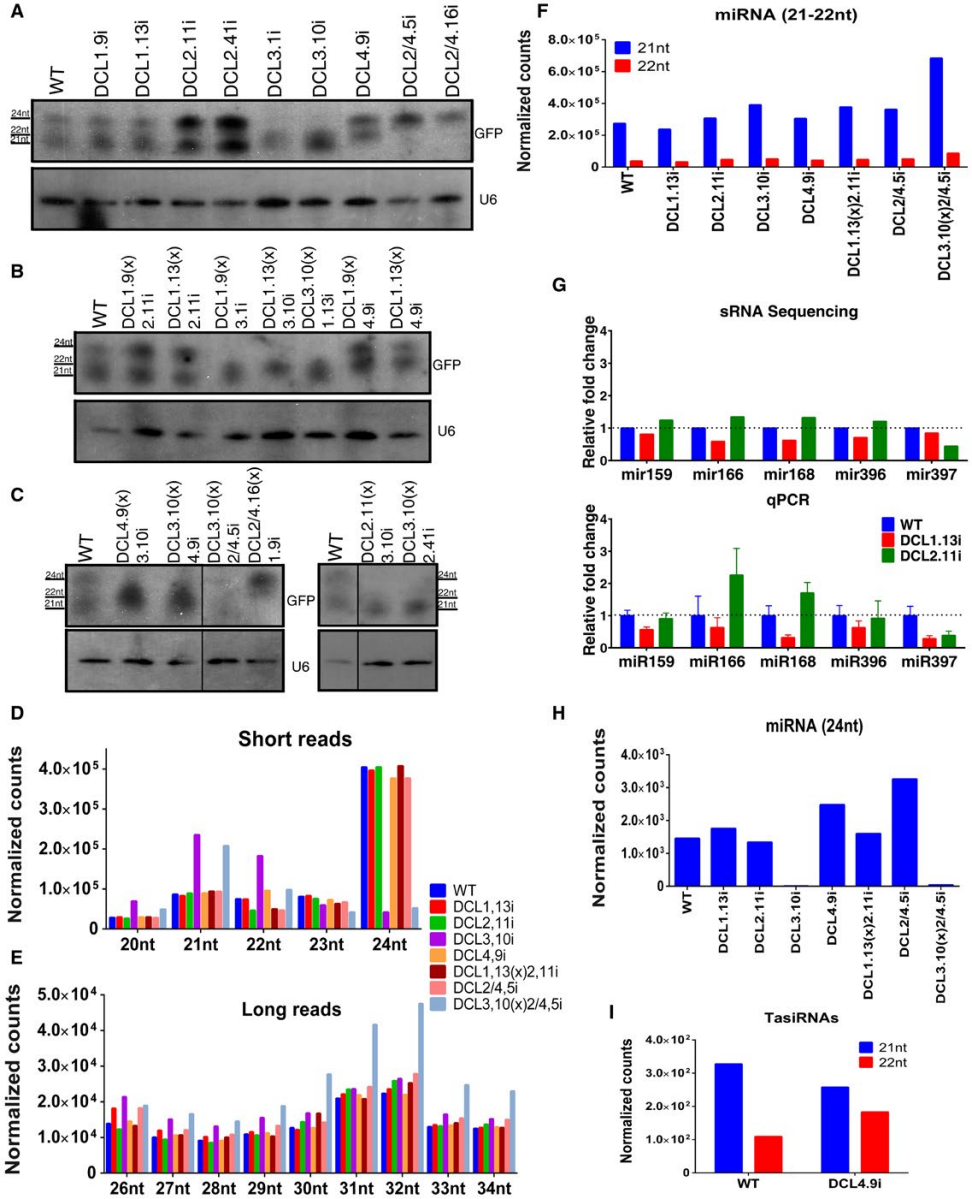
Next‐generation sequencing (NGS) in DCLi and DCLi crossed plants. (A–C) Northern blot analysis of DCLi and DCLi crossed lines agroinfiltrated with green fluorescent protein (GFP) and GFPhp constructs; 21‐, 22‐ and 24‐nucleotide (nt) small RNAs were monitored. U6 was used as internal control. (D, E) Distribution of 20–24‐nt and 26–34‐nt small RNAs in wild‐type (WT), DCL1.13i, DCL2.11i, DCL3.10i, DCL4.9i, DCL1.13(x)2.11i, DCL2/4.5i and DCL3.10(x)2/4.5i. (F, H, I) 21–24‐nt micro‐RNA (miRNA) and trans‐acting small interfering RNA (tasiRNA) levels in the sequenced plant lines. (G) Validation of the bioinformatics analysis with quantitative polymerase chain reaction (qPCR) of miR159, miR166, miR168, miR396 and miR397. [Colour figure can be viewed at wileyonlinelibrary.com]

In order to characterize the small RNA populations of DCLi plant lines, two distinct but complementary methods were selected. First, we performed agroinfiltration experiments to analyse the consequences of DCL down‐regulation in the production of siRNAs processed from a hairpin with green fluorescent protein (GFP) sequences. As shown in Fig. 2A–C, DCL2i plant lines exhibited reduced production of the 22‐nt class, DCL3i of the 24‐nt class and DCL4i of the 21‐nt class, combined with an increase in 22‐ and 24‐nt small RNAs. Similar results were observed when two or three DCL proteins were reduced. For instance, DCL2/4i plants showed the production of only the 24‐nt population. These results suggest that each DCLi plant presents a decrease in the cognate small RNA population depending on the specific down‐regulated DCL.

Next, we performed high‐throughput sequencing of small RNAs from all single and some double/triple DCLi plants (DCL1.13i, DCL2.11i, DCL3.10i, DCL4.9i, DCL1.13(x)2.11i, DCL2/4.5i and DCL3.10(x)2/4.5i). We sequenced a pool of four different plants from one line in a single run for each DCL suppression. Therefore, it cannot be excluded that other transgenic lines could show some differences in the small RNA populations. Eight different libraries were obtained (Table S2, see Supporting Information). First, we investigated the length distribution of sRNAs obtained in the lines analysed. The 24‐nt class was found to be largely predominant in WT, followed by the 21‐ and 22‐nt classes, which correlates with previously published results (Baksa *et al.*, 2015; Kangquan *et al.*, 2015; Xiao *et al.*, 2014).

In the DCL1.13i plant line, a small decrease in the 21‐nt small RNAs was observed, which corresponds to the moderate reduction in DCL1 in this plant line (Dadami *et al.*, 2013; Katsarou *et al.*, 2016) (Fig. 2D; Tables S1 and S3, see Supporting Information). In the DCL2.11i plant line, a 38% decrease in the 22‐nt small RNAs, combined with a small increase in the 21‐nt population, was observed. Plant line DCL3.10i showed a significant increase in the 21‐ and 22‐nt small RNAs (173% and 144%, respectively) and a 90% reduction in the 24‐nt class. Such changes on DCL3 suppression have been reported in other organisms, such as *Solanum lycopersicum* and *A. thaliana* plants, and is probably the result of an increased accessibility of the substrates normally processed by DCL3 by the other DCLs (Blevins *et al.*, 2015; Henderson *et al.*, 2006; Kasschau *et al.*, 2007; Kravchik *et al.*, 2014a). A decrease in the 21‐nt population was not visible in the DCL4.9i plant line as DCL4 is responsible for the production of a limited number of tasiRNAs, but a 28% increase in the 22‐nt population was observed in this line, linked to the role of DCL2 in their generation. When DCL1 and DCL2 were simultaneously reduced, a 33% decrease in the 22‐nt population was observed, combined with a small increase in the 21‐nt class. In DCL2/4.5i plants, a 38% decrease was observed in the 22‐nt population and an 8% increase in the 21‐nt class, which probably corresponds to miRNAs and the activity of DCL1. Finally, when DCL2, DCL3 and DCL4 proteins were simultaneously decreased, an 87% decrease in the 24‐nt population was observed, together with an increase in the 21/22‐nt population. As this increase was smaller than that observed in DCL3.10i plants, we concluded that this increase is partially caused by the activity of DCL2 and DCL4. However, in DC2/4.5i plants, a significant decrease in the 22‐nt class was found, suggesting that the increase in the 21/22‐nt population observed in DCL3.10(x)2/4.5i is probably caused by the activity of DCL1 (Fig. 2D; Table S3). It should be noted that, when comparing the agroinfiltrated small RNA profile and next‐generation sequencing (NGS) profile in DCLi lines (Figs 2A,B and 2D), small differences in the observed populations are detected. This is because, in the northern blot, we detect products corresponding to the probe used, whereas, in NGS, we detect the whole small RNA population.

We also investigated small RNAs from 25 to 34 nt and found a marked increase in this group of siRNAs in DCL3.10(x)2/4.5i plants (Fig. 2E; Table S3). This phenomenon has been described in the *A. thaliana dcl2dcl3dcl4 *mutant and could be correlated with the newly identified P4R2 RNAs (Blevins *et al.*, 2015; Li *et al.*, 2015; Yang *et al.*, 2015).

Next, we focused on the analysis of miRNA populations. As a result of the lack of specific miRNA entries for *N. benthamiana* in mirBASE, we considered only miRNAs identified in three previous publications (Baksa *et al.*, 2015; Kangquan *et al.*, 2015; Xiao *et al.*, 2014) (for details, see Experimental procedures). DCL1i plants had 12.7% less reads for the miRNA set, compared with WT plants, which was in accordance with the limited suppression (~30%) of DCL1 in these plants (Fig. 2F; Table S1). A complete list of the studied miRNAs is presented in Table S4 (see Supporting Information). Five miRNAs were selected for quantitative polymerase chain reaction (qPCR) analysis in these lines in order to validate the NGS analysis. For accurate qPCR analysis, we used three small nucleolar RNAs as reference (U1, U4 and U6). As shown in Fig. 2G, miR159, miR166, miR168, miR396 and miR397 were confirmed to be down‐regulated in DCL1.13i plants. In *Arabidopsis*, all of these miRNAs are involved in important plant processes, such as leaf, root and seed development, as well as stress tolerance (Li and Zhang, 2016; Xian *et al.*, 2018). DCL2i plants showed a small increase in the miRNA population in NGS, which was also verified by qPCR (Fig. 2F,G). In DCL1.13(x)2.11i plants, some miRNAs were found to be decreased compared with WT, probably as a result of reduced levels of DCL1, but some miRNAs showed up‐regulation. This eventually suggests a potential involvement of DCL4 in the production of specific miRNAs in the absence of DCL1 and DCL2, as can be shown for Nbe‐mir154 and Nbt‐mirN79, whose expression was increased in DCL1.13(x)2.11i plants, but decreased in DCL4.9i plants (Table S4). An involvement of DCL4 in miRNA production has already been proposed for *A. thaliana* miR822, miR839 and miR859 (Ben Amor *et al.*, 2009; Rajagopalan *et al.*, 2006). However, a differential expression of pre‐miRNAs in DCL1.13(x)2.11i plants cannot be excluded. In DCL3.10i and DCL3.10(x)2/4.5i plants, miRNAs were increased overall, with the exception of a small number of miRNAs (Table S4), which could be correlated with an increased activity of DCL1 in the absence of DCL3/DCL2/DCL4. Alternatively, in the absence of other DCLs, we have sequenced a better representation of DCL1‐produced small RNAs. Furthermore, both display a prominent reduction in 24‐nt miRNAs (Fig. 2H). Altogether, these results suggest that DCL proteins other than DCL1 can participate in the production of some miRNAs in *N. benthamiana*.

The DCL4i plant line was further used for the analysis of potential tasiRNAs. These small RNAs of 21 nt in length are processed by DCL4 in *Arabidopsis*, tomato and rice (Gasciolli *et al.*, 2005; Henderson *et al.*, 2006; Song *et al.*, 2012; Xie *et al.*, 2005; Yifhar *et al.*, 2012). Although the biogenesis and physiological roles of tasiRNAs have been relatively well documented in *A. thaliana*, there is very limited information on *N. benthamiana *(Krasnikova *et al.*, 2009) tasiRNAs*. *Our analysis showed that DCL4.9i plants presented a modest decrease in known tasiRNAs of 21 nt. In addition, in DCL4.9i, we observed an increase in the number of identified tasiRNAs that have an extra nucleotide compared with their usual sequence, therefore having a size of 22 nt, which suggests a role of DCL2 in the production of these particular tasiRNAs (Fig. 2I). Taken together, our results indicate that DCLi plants show significant differences in their small RNA profiles, correlating with the respective DCL that is suppressed.

### Applications of DCLi plant lines

With the previously described analysis, we were able to show that *N. benthamiana* DCLi plant lines are functionally informative, as the absence of each DCL protein affects the cognate small RNA population and the resulting phenotype in a distinct manner. In order to show the significance and range of applications of these plants, we selected two approaches: protein overexpression and viral infections.

#### Transient overexpression of proteins

**Figure 3:**
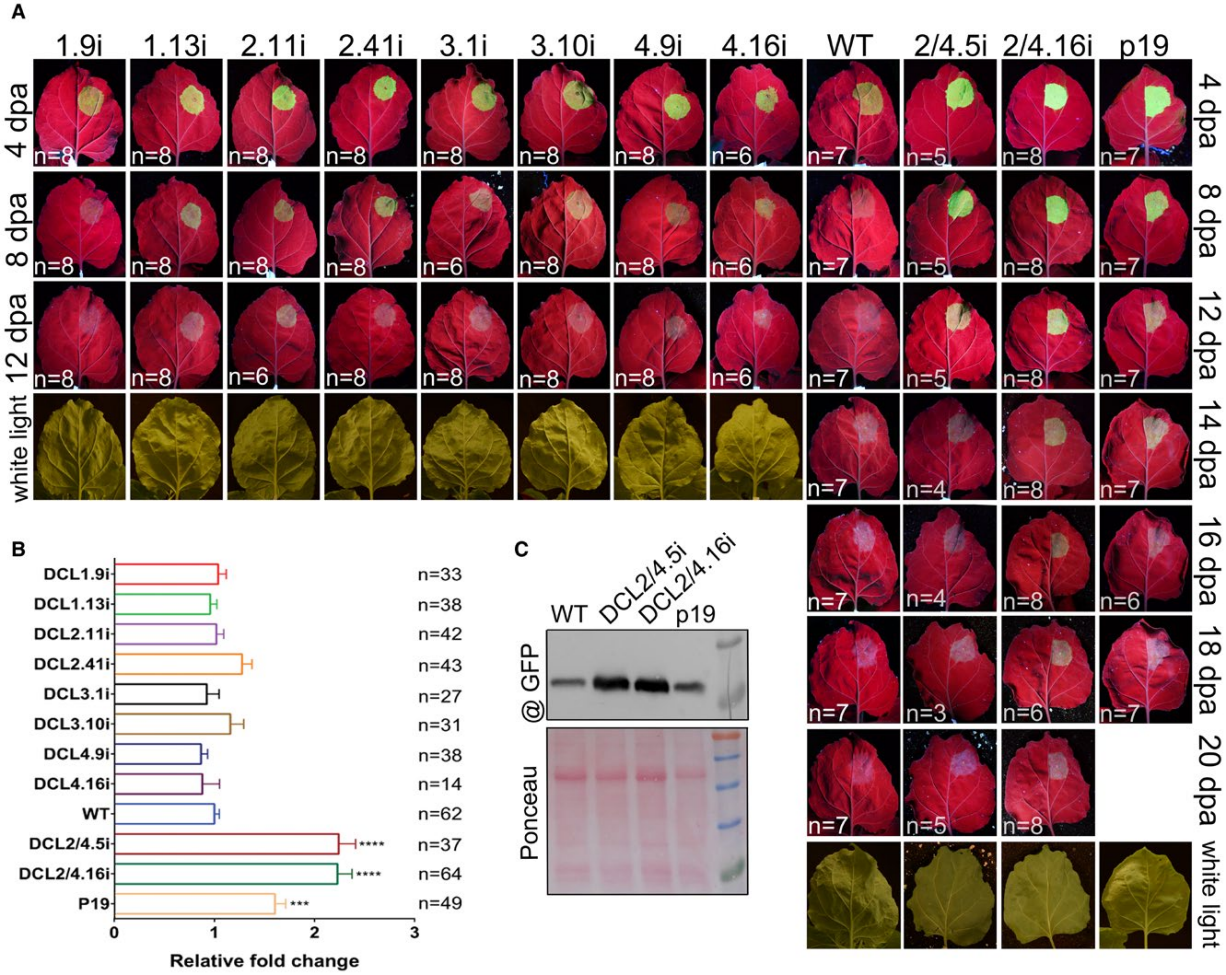
Green fluorescent protein (GFP) expression in DCLi plant lines. (A) Photographs of GFP agroinfiltrated leaves under UV or white light at different time points. ‘*n*’ corresponds to the number of leaves with the same GFP intensity as in the presented photograph. DCLi plants are at the F7 generation, apart from DCL3.1i and DCL3.10i, which are at the F4 generation. (B) Measurement of GFP in DCLi plant line total protein extracts at 4 days post‐agroinfiltration (dpa). ‘*n*’ corresponds to the number of individual leaves tested. One‐way analysis of variance (ANOVA) test was performed with ****P < 0.0001 and ***P < 0.001. (C) Western blot analysis for GFP detection in wild‐type (WT), DCL2/4i and P19 plant lines. Ponceau staining was used as loading control. [Colour figure can be viewed at wileyonlinelibrary.com]

Heterologous protein expression in plants is an interesting choice for the overexpression of molecules for cosmetic, pharmaceutical or other uses (Pua *et al.*, 2017; Rattanapisit *et al.*, 2017; Yang *et al.*, 2017). One important limiting factor in this approach remains the yield of the proteins produced (Desai *et al.*, 2010; Kusnadi *et al.*, 1997). As RNA silencing is a major obstacle in the overexpression of exogenous proteins in plant tissues, we tested whether our DCLi lines, which are expected to be impaired in RNA silencing, could be useful tools to increase transient protein expression. To this end, we performed agroinfiltration experiments using GFP as a reporter (Ziemienowicz, 2001). WT and p19 plants overexpressing the suppressor of silencing from *Cymbidium ringspot virus* (Family, *Tombusviridae*; Genus, *Tombusvirus*) were used as controls. Experiments were performed at least three times and the number (*n*) of observed leaves is presented in each photograph. As shown in Fig. 3, in all DCLi plants, GFP is expressed. However, in DCL2/4.5i plants, GFP fluorescence can be observed as late as 16 days post‐agroinfiltration (dpa), similar to plants expressing the p19 silencing suppressor, and, in DCL2/4.16i plants, GFP can be visually detected even at 20 dpa (Fig. 3A). This correlates with a greater reduction in DCL2 and DCL4 in DCL2/4.16i plants compared with the DCL2/4.5i line (Dadami *et al.*, 2013; Katsarou *et al.*, 2016). GFP fluorescence was also measured in crude extracts at 4 dpa, which is the most used time point in agroinfiltration experiments. As shown in Fig. 3B, both DCL2/4.5i and DCL2/4.16i plant lines expressed GFP 2.2‐fold more strongly than did WT plants, which was even higher than that observed for p19‐expressing plants (1.6‐fold change). Differences in GFP expression were also confirmed by western blotting (Fig. 3C). Furthermore, agroinfiltration of GFP together with p19 in the DCL2/4.16i background did not provide any further improvement of the GFP signal, indicating that a maximum expression of GFP was reached in DCL2/4i plants (Fig. S2, see Supporting Information). In the rest of the DCLi plant lines, no significant differences in their capacity to express GFP were observed, confirming that, in *N. benthamiana*, it is the combined action of DCL2 and DCL4 proteins that contributes to sense post‐transcriptional gene silencing and therefore limits the capacity of the system to overexpress proteins. In DCLi crosses, we could not observe significant differences in GFP expression capacity or timing of expression compared with WT, apart from the triple knockdown plants DCL2‐DCL3‐DCL4 and DCL1‐DCL2‐DCL4, where we observed similar results to the DCL2/4i plant lines (Fig. S3, see Supporting Information).

Taken together, these results indicate that the DCL2/4i plant lines can be a useful tool for the *in planta* overexpression of exogenous proteins. Furthermore, DCL2/4i plants, apart from increased branching, do not show phenotypic defects, unlike p19 plants which exhibit a relatively severe phenotype, making the agroinfiltration applications more demanding (Kontra *et al.*, 2016; Siddiqui *et al.*, 2008; Stav *et al.*, 2010).

##### DCLi plant lines and viral infections

In order to evaluate the role of *N. benthamiana* DCL proteins in antiviral responses and, at the same time, estimate the potential of these lines for the study of host–virus interactions, we performed infection with three RNA viruses. We opted for viruses which (i) are natural infectious agents of *N. benthamiana*, (ii) have an active suppressor of silencing, and (iii) are well studied in other organisms, as a proof of principle that DCLi plant lines, because of their higher susceptibility, can increase viral accumulation and, consequently, detection efficiency.

**Figure 4:**
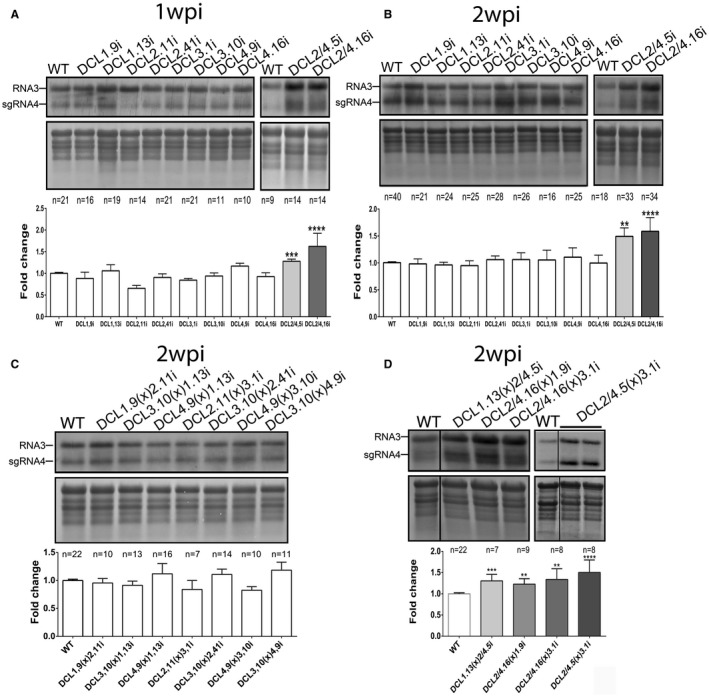
*Cucumber mosaic virus* (CMV) infections in DCLi and DCLi crossed plants. Representative northern blots of CMV DCLi plant lines infected for 1 week post‐infection (wpi) (A) or 2 wpi (B), and DCLi crossed plants infected for 2 wpi (C, D). The CMV probe that recognizes part of the transcript of the coat protein (CP) was used (RNA3 and sgRNA4). Methylene blue staining was used as loading control. Graphs represent measurements performed with Quantity One 4.4.1 (Biorad). ‘*n*’ denotes the number of individual tested plants. Statistical significance was tested with one‐way analysis of variance (ANOVA) and Student’s *t*‐test. The *P* value was set at **P < 0.01, ***P < 0.001 and ****P < 0.0001. WT, wild‐type.

We infected DCLi and F1 crossed plants with *Cucumber mosaic virus* (CMV), a well‐studied virus of significant economic impact infecting an important number of hosts (reviewed in Jacquemond, 2012). CMV (Family, *Bromoviridae*; Genus, *Cucumovirus*) is a multipartite (+) single‐stranded RNA (ssRNA) virus (RNA1, RNA2 and RNA3) encoding five proteins. An additional protein (2b) is produced by the subgenomic RNA4A, which constitutes the suppressor of silencing (Jacquemond, 2012). Infections were made using CMV^GR21^, a strain isolated in northern Greece belonging to subgroup I, causing severe phenotypes (Kalantidis *et al.*, 2002). We performed three independent experiments, collected tissue at 1 and 2 weeks post‐infection (wpi) and analysed the infection by northern blots. The number of tested plants is indicated in each case with an ‘*n*’ (Figs 4 and S4A, see Supporting Information). We found increased virus titre and symptoms in DCL2/4.5i and DCL2/4.16i plants at both 1 and 2 wpi compared with WT infected plants. In contrast, no significant alteration was observed in single DCL2i or DCL4i plants, suggesting a redundancy of DCL2 and DCL4 in CMV defence. In addition, we performed infections in DCLi crosses (three independent experiments) and observed enhanced viral titre in all crosses of the DCL2/4i background (DCL2/4.5(x)3.1i, DCL2/4.16(x)3.1i, DCL2/4.16(x)1.9i and DCL1.13(x)2/4.5; Fig. 4C), further verifying the redundancy between DCL2 and DCL4.

**Figure 5:**
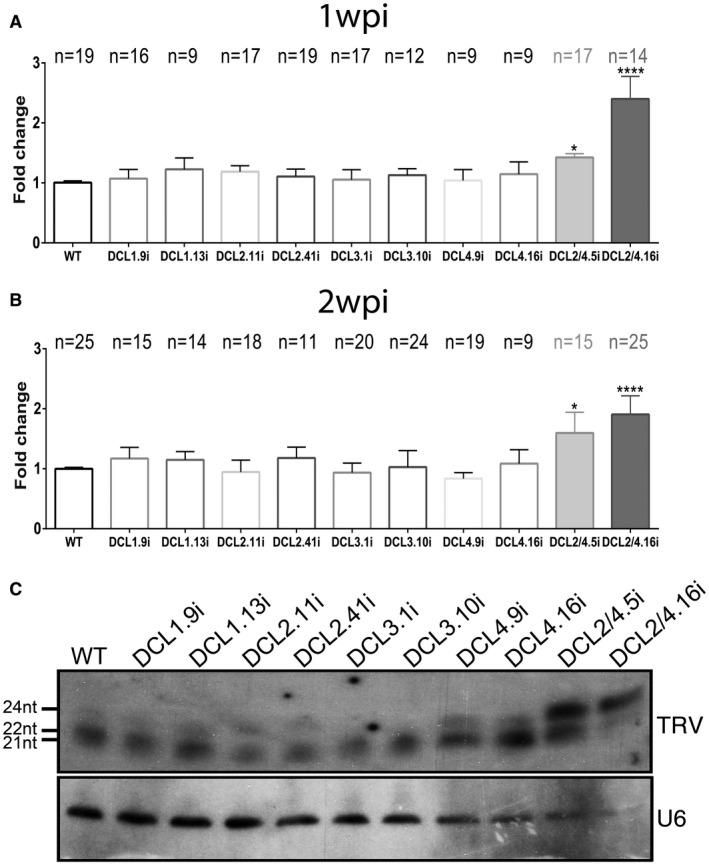
*Tobacco rattle virus* (TRV)‐infected DCLi plants. Graphs representing measurements performed with Quantity One 4.4.1 (Biorad) in northern blots at 1 week post‐infection (wpi) (A) and 2 wpi (B). Statistical analysis was performed with one‐way analysis of variance (ANOVA) test, and the *P* value was set at *P < 0.05 and ****P < 0.0001. ‘*n*’ represents the number of individual measured plants. (C) Northern blot analysis of TRV vsiRNA from 2 wpi DCLi infected plants. U6 was used as loading control. WT, wild‐type.

The second virus studied was *Tobacco rattle virus* (TRV). TRV (Family, *Virgaviridae*; Genus, *Tobravirus*) is a bipartite (+) ssRNA virus (RNA1 and RNA2) with the suppressor of silencing (16K) encoded by RNA1. This virus has been utilized extensively for research purposes as it has been modified to be used as a viral vector for protein expression and VIGS experiments (Macfarlane, 2010). We performed infections with the TRV^PpK20^‐GFP strain to facilitate the visualization of infection using UV together with northern blots at 1 and 2 wpi. We performed three independent experiments, with ‘*n*’ indicating the number of plants that were infected and taken into consideration on analysis. TRV levels were increased in DCL2/4i plants at both 1 and 2 wpi (Fig. 5), which correlated with severe symptoms compared with WT plants (Fig. S4B). Furthermore, analysis of TRV sRNAs by northern blot showed a positive correlation between DCL suppression and the reduction in its cognate small RNA population (Fig. 5C). In the absence of DCL3 protein, the 24‐nt small RNA class of TRV decreased significantly. In DCL4i plants, 21‐nt levels were minimized, combined with an increase in the 22‐ and 24‐nt populations, suggesting increased substrate availability for DCL2 and DCL3 proteins in the absence of DCL4. Finally, when both DCL2 and DCL4 were decreased, a significant increase in the 24‐nt viral small RNA was observed, presumably as a result of DCL3 activity. An increase in GFP fluorescence was also observed in DCL2/4i roots, suggesting a role for DCL2 and DCL4 in the systemic spread of TRV in both leaf and root tissues (Fig. S5, see Supporting Information). This observation also suggests that DCLi plants may be an excellent tool for root studies as the RNAi mechanism is altered in the roots of these plants.

**Figure 6:**
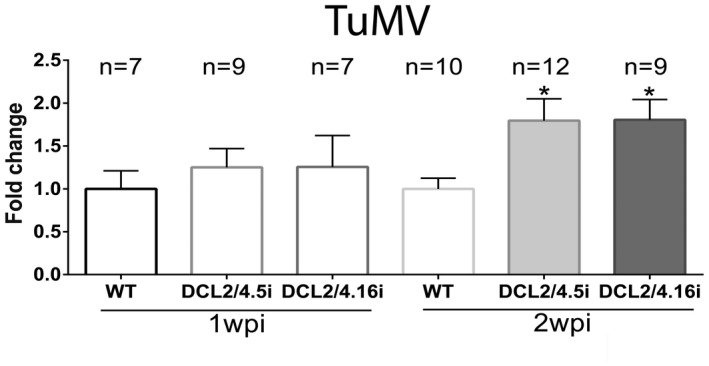
*Turnip mosaic virus* (TuMV)‐infected DCL2/4i plants. Graph representing measurements with Quantity One 4.4.1 (Biorad) from three independent experiments of semi‐quantitative polymerase chain reaction (PCR) in DCL2/4i plants infected with TuMV for 1 or 2 weeks post‐infection (wpi). ‘*n*’ shows the number of individual tested plants. Student’s *t*‐test was performed with *P < 0.05.

Finally, we infected DCL2/4i plants in three independent experiments with *Turnip mosaic virus* (TuMV^FKD004J^), an economically important virus infecting a wide range of monocotyledons and dicotyledons worldwide (Yasaka *et al.*, 2017). TuMV (Family, *Potyviridae*; Genus, *Potyvirus*) contains a unique (+) ssRNA producing a polyprotein with at least 10 mature proteins, among them HC‐Pro, a suppressor of silencing (Anandalakshmi *et al.*, 1998). Virus levels were analysed in TuMV‐infected DCL2/4.5i and DCL2/4.16i plants by semi‐quantitative PCR. No significant difference in viral levels was observed at 1 wpi, whereas, at 2 wpi, a *c*. 80% increase in viral RNA was monitored in both plant lines (Fig. 6). This was also accompanied by enhanced symptoms (Fig. S4C).

**Figure 7:**
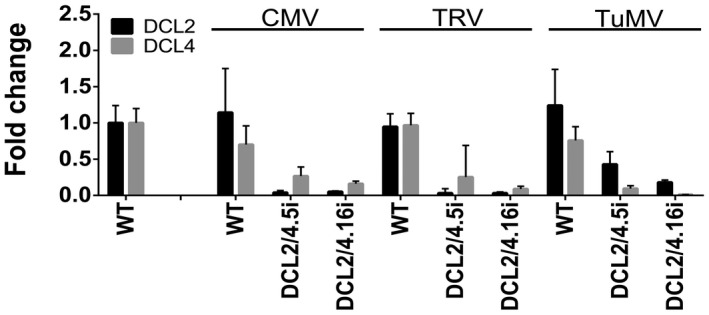
DCL2 and DCL4 levels in *Cucumber mosaic virus* (CMV)‐, *Tobacco rattle virus* (TRV)‐ and *Turnip mosaic virus* (TuMV)‐infected DCL2/4i plants. Quantitative polymerase chain reaction (qPCR) experiments for the detection of endogenous DCL2 and DCL4 transcripts in CMV‐, TRV‐ or TuMV‐infected DCL2/4.5i and DCL2/4.16i plant lines at 2 weeks post‐infection (wpi).

As all three viruses, as mentioned, contain suppressors of silencing, we reasoned that their function could influence the processing of expressed hairpin in DCLi infected plant lines. To this end, we performed qPCR experiments addressing the levels of targeted DCLs in DCL2/4i plant lines. As shown in Fig. 7, the infected plant lines retained a significant reduction in the targeted DCL, showing the effectiveness of RNAi suppression, even in the presence of the different suppressors of silencing proteins.

In all three cases, we were able to show an overall increase in viral titre when both DCL2 and DCL4 proteins were decreased, which was not observed when DCL2 or DCL4 alone was decreased. This is in accordance with previous proposals in *Arabidopsis* (Bouché *et al.*, 2006; Deleris *et al.*, 2006; Diaz‐Pendon *et al.*, 2007; Donaire *et al.*, 2008; Kung *et al.*, 2014; Lewsey and Carr, 2009). To our knowledge, this is the first time that the role of each DCL on CMV, TRV and TuMV infection in *N. benthamiana* has been assessed.

In conclusion, we present here *N. benthamiana* plants efficiently suppressed for each and every DCL protein, as well as their combinations. In addition to recent reports of other NbDCLi plants (Andika *et al.*, 2015; Matsuo and Matsumura, 2017; Qin *et al.*, 2017), we have gone a step further and performed a detailed phenotypic and molecular analysis, making these DCLi plant lines an interesting and valuable tool for research or commercial purposes.

## Experimental Procedures

### Plant materials and photographs

DCLi plant lines have been described previously (Dadami *et al.*, 2013). Crosses were made with a standard crossing protocol. DCLi single and crossed plant lines were followed until the F10 and F4 generations, respectively. The P19 plant line was kindly provided by Dr P. Tavazza. Plant photographs were taken using a Nikon D5100 camera (Nikon, Tokyo, Japan). Seed and flower photographs were taken using a Stemi DV4 stereoscope (Zeiss, Jena, Germany).

### Scanning electron microscopy

For scanning electron microscopy, samples were fixed in 2% glutaraldehyde, 2% paraformaldehyde in 0.1 m sodium cacodylate buffer, pH 7.4, for 30 min at 4 °C, washed in this buffer twice, post‐fixed in 1% aqueous OsO_4_ for 30 min at 4 °C and rinsed as above. Samples were dehydrated through a graded ethanol series, 30%–50%–70%–90%–100% at 4 °C, followed by dry alcohol in room temperature. Dehydrated samples were critical point dried (Baltec, Los Angeles, California, USA CPD 030) and mounted on stubs prior to sputter coating with 10‐nm‐thick gold (Baltec SCD 050). Observations were carried out using a scanning electron microscope (JEOL, model JSM‐6390LV‐, Tokyo, Japan) at an operating voltage of 15 kV.

### Small RNA sequencing

Total RNA was extracted from eight leaves of 10‐leaf‐stage plants from four different plants of each sequenced line, as described previously (Katsarou *et al.*, 2016). After treatment with DNAseI (Roche Diagnostics, Basel, Switzerland) for 30 min at 37 °C, samples were cleaned with phenol–chloroform. Integrity was confirmed in denatured agarose gel (1% agarose, 0.7% formaldehyde). Small RNA sequencing and library construction were performed by GenXpro (http://genxpro.net/). FASTQ file generation, demultiplexing and adapter removal were performed by GenXpro.

### Bioinformatic analysis

Sequences originating from the DCL hairpins were eliminated by aligning raw sequencing data to the hairpin sequencing using bowtie (Langmead *et al.*, 2009). A mismatch parameter threshold of 1 was used in this preprocessing step. Normalization was performed using the following equation: normalized count = count/total count × 1 000 000. Length distributions were calculated by summing normalized counts for each individual sample.

miRNA data analysis was retrieved directly from the cleaned FASTQ files using in‐house scripts, and sequences were annotated by blastn (http://www.ncbi.nlm.nih.gov/BLAST/) against mirBASE (http://www.mirbase.org/) and the *Nicotiana* genome (http://www.ncbi.nlm.nih.gov/genome/?term=CBMM01). However, as no specific annotation of *N. benthamiana* miRNA is available, we considered only miRNA sequences from already published research (Baksa *et al.*, 2015; Kangquan *et al.*, 2015; Xiao *et al.*, 2014). Xiao *et al.* (2014) have adopted the ‘Nbe’ prefix for their predicted miRNAs, Kangquan *et al.* (2015) use ‘Nbt’ and Baksa *et al.* (2015) assign ‘Nb’. We selected reads with 100% homology to the miRNA sequence derived from these three studies. As the number of reads for these miRNAs is inferior to 10^6^, we performed normalization of miRNAs using the following equation: normalized counts = count × mean of all libraries/count of each library. In total, 620 unique miRNA sequences were used for the analysis, 180 of which were ‘star’ sequences. This analysis was performed using RSEM (Li and Dewey, 2011). All raw data have been uploaded to the European Nucleotide Archive (http://www.ebi.ac.uk/ena/data/view/PRJEB25121).

### Quantitative PCR

For miRNA, 500 ng of DNAseI‐treated RNA (Roche Diagnostics) from four independent plants was reverse transcribed with RT polymerase (Minotech Biotechnology, Heraklion, Crete, Greece) using deoxyribonucleotide triphosphate(s) (dNTPs) (Invitrogen/Thermo Fisher Scientific, Waltman, Massachusetts, USA) and 0.5 μm of the adequate reverse RT primer designed according to Chen *et al.* (2005) and Varkonyi‐Gasic *et al.* (2007) (Table S5, see Supporting Information). The mix was heated at 80 °C for 5 min. After the addition of the manufacturer’s buffer, 5 mm dithiothreitol (DTT) and 40 units recombinant ribonuclease inhibitor (RRI) (Takara, Kusatsu, Japan, the mix was incubated for 30 min at 16 °C, 60 min at 55 °C and 15 min at 72 °C. qPCR amplification was carried out in a CFX CONNECT™ apparatus (Biorad, Hercules, California, USA) using a Kapa SYBR Fast qPCR Kit (Kapa Biosystems, Basel, Switzerland), as described by the manufacturer. The used primer melting temperatures (Tms) are described in Table S5. As internal reference we used three different nucleolar small RNAs (U1, U4 and U6) which are stable in the tested conditions according to NormFinder and BestKeeper algorithms (Andersen *et al.*, 2004; Pfaffl *et al.*, 2004; Turner *et al.*, 2013). qPCR for DCL has been described previously (Katsarou *et al.*, 2016). L23, PP2A and FBOX were used as reference genes; all three tested stable on CMV, TRV and TuMV infections according to NormFinder and BestKeeper (Andersen *et al.*, 2004; Liu *et al.*, 2012; Pfaffl *et al.*, 2004). Primers, including TuMV already published primers, are presented in Table S5 (Sanchez *et al.*, 2003).

### RNA extraction and northern blots

Total RNA extraction from young leaves has been described previously (Katsarou *et al.*, 2016). Large RNAs (4 μg) were separated in 1% agarose/0.7% formaldehyde gel, whereas small RNAs (20 μg) were separated in 17% polyacrylamide (38 : 2) gel. Both were transferred to 0.2‐μm Nylon membrane (Whatman, GE Healthcare, Chicago, Illinois, USA). Probing for large RNAs was performed using digoxigenin (DIG)‐labelled probes (Roche Diagnostics). For CMV detection, DIG‐labelled RNA was produced by T7 *in vitro* transcription (Takara) from pBluescript‐CMV‐gr21/*Bam*HI plasmid (Kalantidis *et al.*, 2002). For TRV detection, DIG‐labelled RNA was produced by T7 *in vitro* transcription (Takara) from plasmid pGEM‐TRV/*Pst*I, created for this purpose using the primers presented in Table S5. For the probing of small RNAs, 100 ng of a GFP or TRV PCR product was produced from plasmids pBIN‐mGFP4 and pGEM‐TRV2, respectively (Table S5). PCR products were labelled with [α‐^32^P]CTP using Klenow (Minotech). Hybridization was performed at 45 °C as described previously (Dadami *et al.*, 2013).

### Agroinfiltrations

For agroinfiltration experiments, the following constructs were used: pBIN‐mGFP4 (kindly provided by Dr Haseloff; Haseloff *et al.*, 1997), FGC5941‐hpGFP (Kościańska *et al.*, 2005), pART27 (Gleave, 1992) and 35SP19‐CymRSV (kindly provided by Dr Burgyan), all in the C58C1 *Agrobacterium* strain. The protocol for agroinfiltration experiments has been described previously (Helm *et al.*, 2011; Kościańska *et al.*, 2005). An optical density at 600 nm (OD_600_) of 0.1 was used in these experiments. Monitoring of GFP expression was performed with a hand‐held 100‐W long‐wavelength UV lamp (B1000AP; Ultraviolet Products, Upland, California, USA).

### Western blot

For western blot analysis, 100 μg of tissue was dissolved in RIPA buffer, sonicated for 3 × 20 s and separated for 1 min on ice (VSX130, Sonics and Materials Inc, Newtown, Connecticut, USA). After centrifugation, total proteins were quantified with the DC™ protein assay (Biorad), and 50 μg were loaded onto a 12% polyacrylamide gel. Transfer to Amersam Protran (0.2 μm) membrane was performed, followed by Ponceau staining and anti‐GFP (Minotech Biotechnology) plotting. Films were developed with ECL Western Blotting substrate (Pierce, Waltham, Massachusetts, USA) in Chemidoc™ XRS (Biorad).

### GFP measurements

GFP measurements were performed as described previously with small modifications (Vogler *et al.*, 2008). Leaf discs were ground in liquid nitrogen with a pestle, and an extraction buffer [62.5 mm Tris‐HCl, pH 6.8, 1% sodium dodecylsulfate (SDS), 20% glycerol] was added. After centrifugation (5 min/13.000g/4 ºC), an equal volume of extraction buffer lacking SDS was added, and samples were measured in 96‐well plates using a Synergy HTX (Biotek, Winooski, Vermont, USA) apparatus (excitation, 485 nm; emission, 520 nm). The assessment of protein levels was performed using Nanodrop measurements (Thermo Fisher Scientific, Waltman, Massachusetts, USA), and used for normalizations.

### Infections

Mechanical infection on the same leaf numbers using carborundum (Prolabo, VWR, Radnor, Pennsylvania, USA) was performed for all tested viruses. The same numbers of young leaves were collected at the adequate time point for each experiment. TRV^PpK20^‐GFP was kindly provided by Dr McFarlane (MacFarlane and Popovich, 2000). Greek strains CMV^GR21^ and TuMV^FKD004J^ were kindly provided by Dr Nikolaos Katis and Dr Maliogka Varvara I (Kalantidis *et al.*, 2002; Ohshima *et al.*, 2007).

### Software

Figures were assembled using Photoshop CS5 (Adobe Systems Incorporated, California, USA). ImageJ was used for the measurement of trichomes and the number and size of stomata. Quantity One 4.4.1 (Biorad) was used for northern blot quantifications, and GraphPad Prism6 (GraphPad Software, San Diego, California, USA) for graph representations and statistical analysis.

## Supporting information


**Fig. S1** ****Scanning electron microscopy (SEM) in leaves of DCLi and DCLi crossed plants. (A) Photographs of trichomes and stomata of DCLi plant lines. Stars indicate positions of stomata. (B, D) Measurements with FiJi of trichrome/stomata number and stomatal size. Statistical analysis was performed with unpaired Student’s *t*‐test with *P < 0.05, **P < 0.01 and ***P < 0.001. (C) Single stomata of DCL4.9i, DCL4.16i and DCL4.9(x)3.10i plant lines.

**Fig. S2** ****GFP/pART27 and GFP/P19 agroinfiltrations in wild‐type (WT) and DCL2/4.16i plant line at different time points. Photographs taken under UV and white light.

**Fig. S3** ****Green fluorescent protein (GFP) agroinfiltrations in DCLi crossed plants at different time points. Photographs taken under UV and white light. ‘*n*’ represents the number of leaves showing the same fluorescence as that represented in the figure.

**Fig. S4** ****Plant phenotype in DCL2/4i plant lines infected with *Cucumber mosaic virus* (CMV) (A), *Tobacco rattle virus* (TRV) (B) or *Turnip mosaic virus* (TuMV) (C).

**Fig. S5** *Tobacco rattle virus* (TRV)‐infected roots in wild‐type (WT) and DCL2/4i plant lines. Photographs taken under UV and white light.

**Table S1** ****Transcript levels of DCLi single and crossed plant lines (adapted from Katsarou *et al.*, 2016).

**Table S2** ****Small RNA deep sequencing libraries.

**Table S3** ****Length (nucleotide, nt) distribution of reads mapped in the *Nicotiana benthamiana* genome (normalized data).

**Table S4** ****Expression levels of micro‐RNAs (miRNAs) in the tested conditions compared with the wild‐type (WT) condition.

**Table S5** ****Primers used in this study.
