# Supplementary figures and images for: DCL‐suppressed Nicotiana benthamiana plants: valuable tools in research and biotechnology
Source: Mol Plant Pathol. 2018 Dec 19;20(3):432–46. doi: 10.1111/mpp.12761 (PMC6637889; doi:10.1111/mpp.12761)

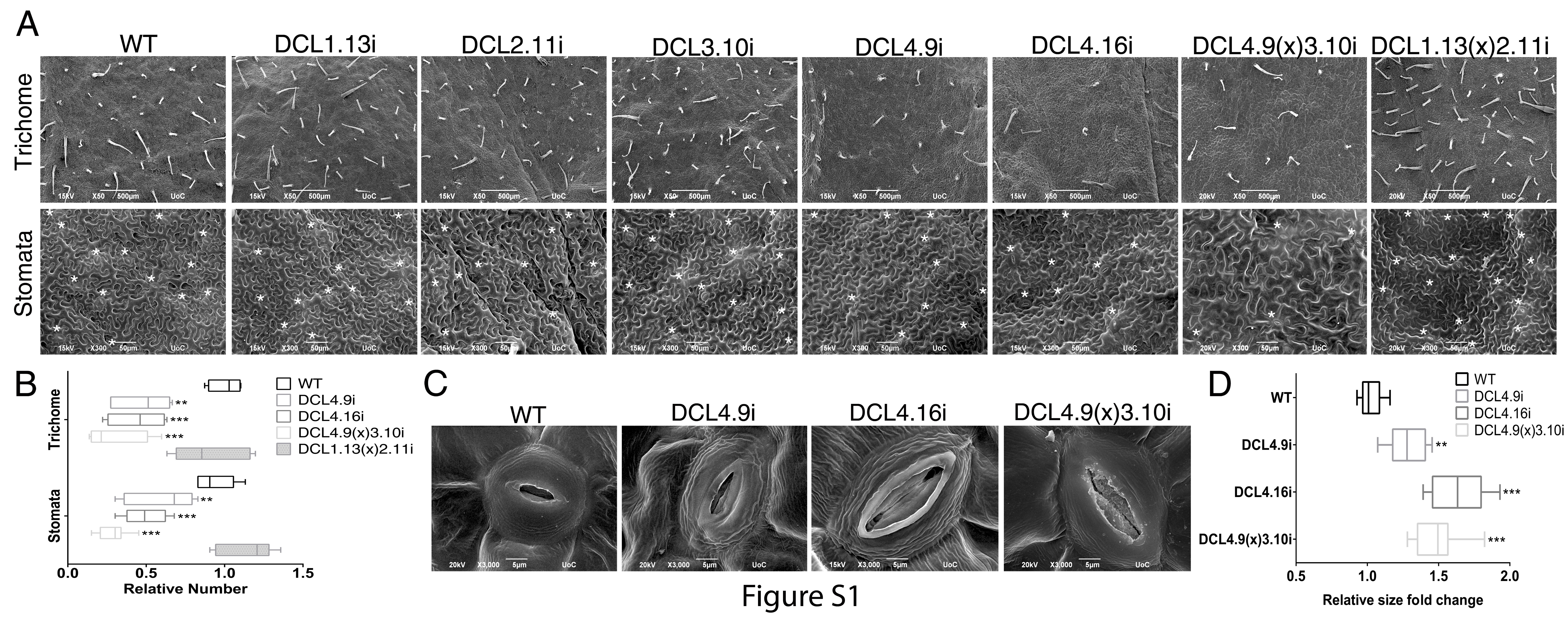

Supplement: Supplementary file 1 — Fig. S1 Scanning electron microscopy (SEM) in leaves of DCLi and DCLi crossed plants. (A) Photographs of trichomes and stomata of DCLi plant lines. Stars indicate positions of stomata. (B, D) Measurements with FiJi of trichrome/stomata number and stomatal size. Statistical analysis was performed with unpaired Student’s t‐test with *P < 0.05, **P < 0.01 and ***P < 0.001. (C) Single stomata of DCL4.9i, DCL4.16i and DCL4.9(x)3.10i plant lines. [file MPP-20-432-s001.jpg]

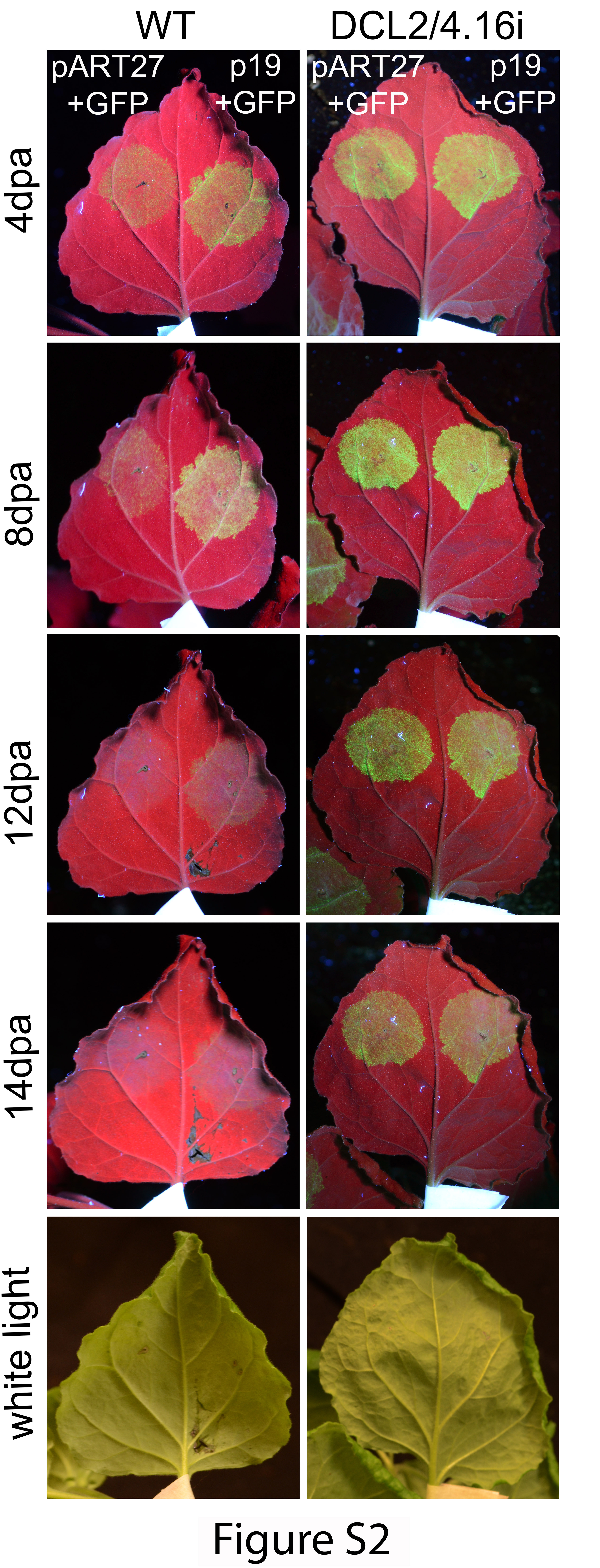

Supplement: Supplementary file 2 — Fig. S2 GFP/pART27 and GFP/P19 agroinfiltrations in wild‐type (WT) and DCL2/4.16i plant line at different time points. Photographs taken under UV and white light. [file MPP-20-432-s002.jpg]

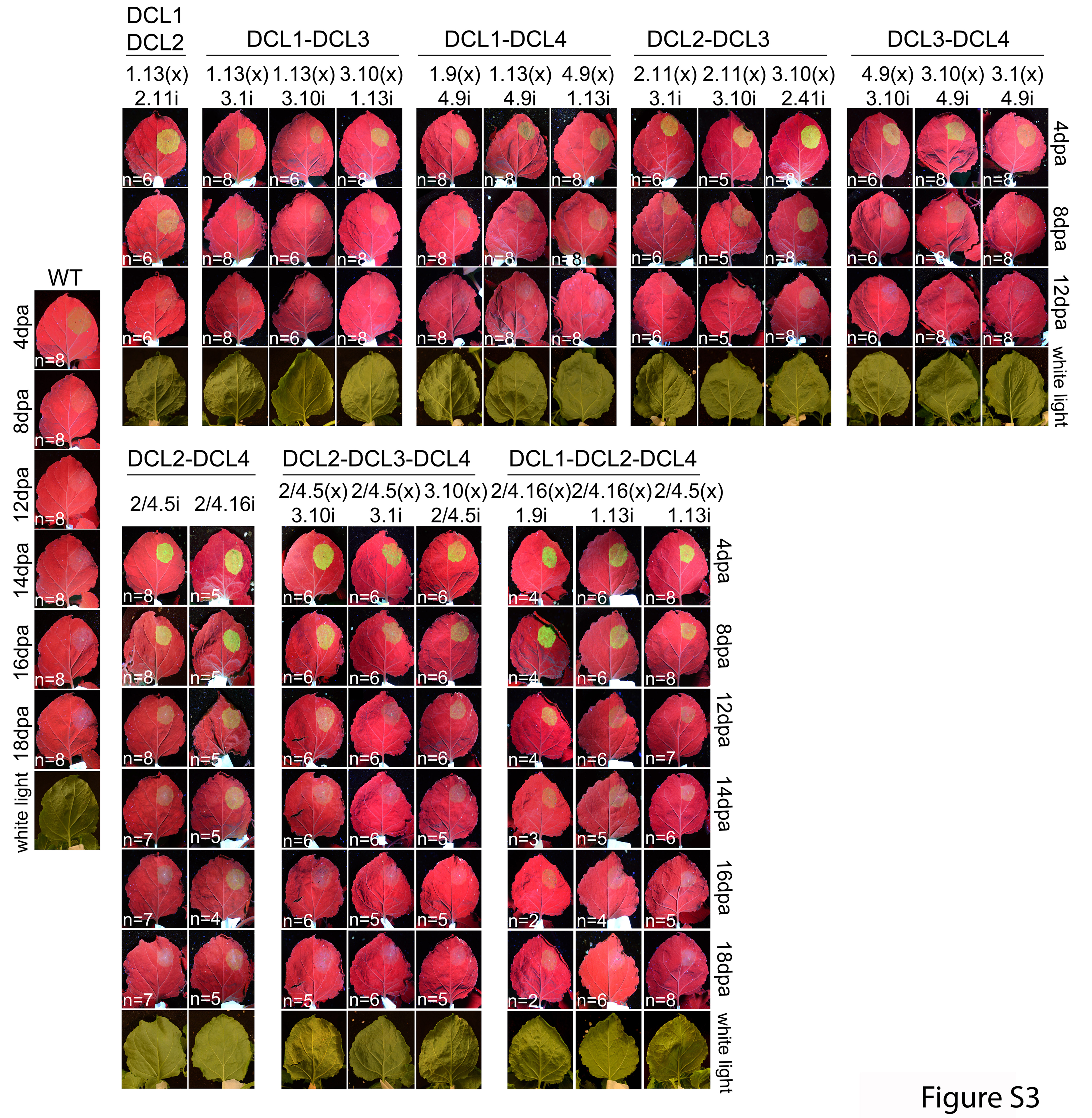

Supplement: Supplementary file 3 — Fig. S3 Green fluorescent protein (GFP) agroinfiltrations in DCLi crossed plants at different time points. Photographs taken under UV and white light. ‘n’ represents the number of leaves showing the same fluorescence as that represented in the figure. [file MPP-20-432-s003.jpg]

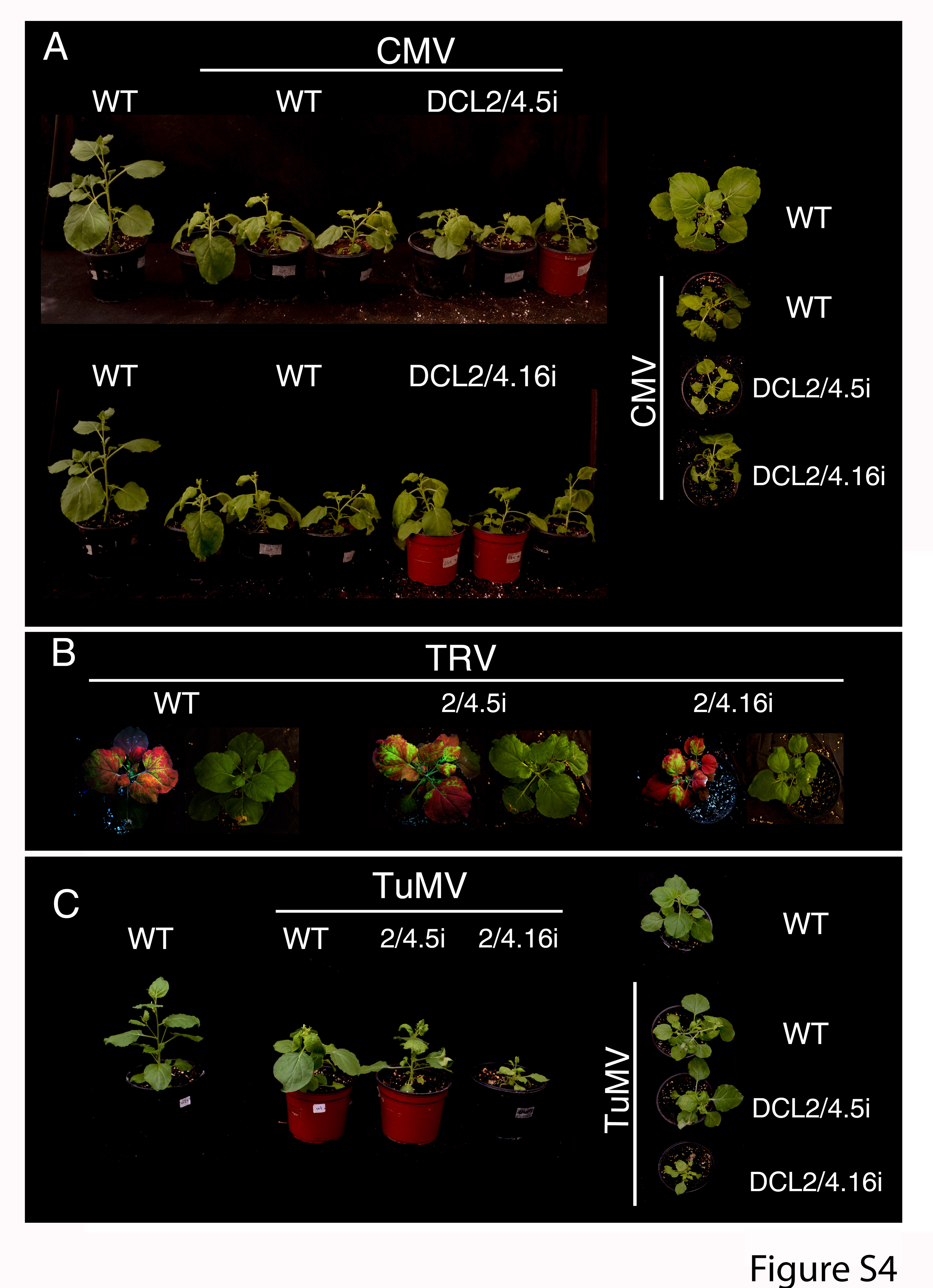

Supplement: Supplementary file 4 — Fig. S4 Plant phenotype in DCL2/4i plant lines infected with Cucumber mosaic virus (CMV) (A), Tobacco rattle virus (TRV) (B) or Turnip mosaic virus (TuMV) (C). [file MPP-20-432-s004.jpg]

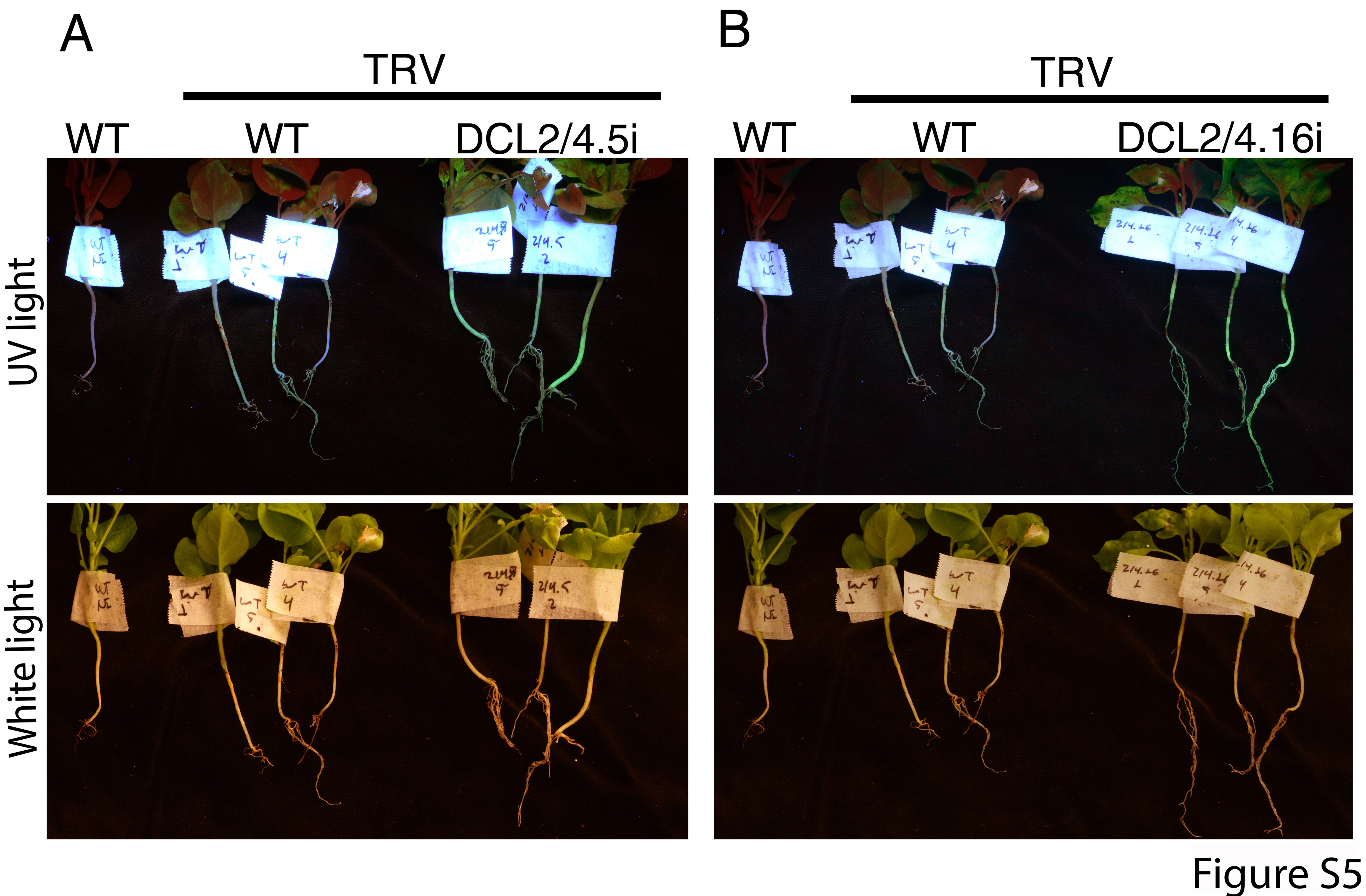

Supplement: Supplementary file 5 — Fig. S5 Tobacco rattle virus (TRV)‐infected roots in wild‐type (WT) and DCL2/4i plant lines. Photographs taken under UV and white light. [file MPP-20-432-s005.jpg]
