# Supplementary material for: DCL‐suppressed Nicotiana benthamiana plants: valuable tools in research and biotechnology
Source: Mol Plant Pathol. 2018 Dec 19;20(3):432–46. doi: 10.1111/mpp.12761 (PMC6637889; doi:10.1111/mpp.12761)
Supplement: Supplementary file 6 — Table S1 Transcript levels of DCLi single and crossed plant lines (adapted from Katsarou et al., 2016). [file MPP-20-432-s006.docx]

|  | **DCL1** | **DCL2** | **DCL3** | **DCL4** |
| --- | --- | --- | --- | --- |
| **DCLi single plant lines** |  |  |  |  |
| **WT** | 1 +/- 0.186 | 1 +/- 0.07 | 1 +/- 0.144 | 1 +/- 0.148 |
| **DCL1.9i** | 0.676 +/- 0.053 | 1.085 +/- 0.072 | 0.837 +/-0.148 | 0.725 +/- 0.193 |
| **DCL1.13i** | 0.618 +/- 0.056 | 1.083 +/- 4.87E-02 | 0.843 +/- 0.292 | 0.84 +/- 4.93E-02 |
| **DCL2.11i** | 1.094 +/- 0.128 | 0.137 +/- 1.52E-02 | 1.147 +/- 0.379 | 0.753 +/- 0.26 |
| **DCL2.41i** | 1.053 +/- 0.121 | 0.072 +/-6.39E-02 | 1.172 +/- 4.77E-02 | 0.796 +/- 0.102 |
| **DCL3.1i** | 0.958 +/- 0.091 | 0.9 +/- 5.60E-02 | 0.346 +/- 0.07 | 0.949 +/- 0.118 |
| **DCL3.10i** | 1.132 +/- 0.106 | 0.994 +/- 4.65E-02 | 0.079 +/- 4.79E-02 | 0.895 +/- 0.085 |
| **DCL4.9i** | 0.912 +/- 0.1 | 1.115 +/- 9.40E-02 | 0.783 +/- 0.125 | 0.314 +/- 0.073 |
| **DCL4.16i** | 0.972 +/- 3.13E-02 | 0.906 +/- 8.00E-02 | 1.151 +/- 0.125 | 0.154 +/- 1.97E-02 |
| **DCL2/4.5i** | 1.139 +/- 0.147 | 0.022 +/- 0.009 | 1.005 +/- 0.355 | 0.034 +/- 0.008 |
| **DCL2/4.16i** | 0.819 +/- 0.209 | 0.001 +/- 8.92 E-05 | 0.850 +/-0.490 | 0.015 +/- 0.008 |
|  |  |  |  |  |
| **DCLi crossed plant lines (F1)** |  |  |  |  |
| **DCL1.13(x)2.11i** | 0.72 +/- 6.70E-02 | 0.071 +/- 1.21E-02 | 0.957 +/- 0.194 | 0.723 +/- 0.197 |
| **DCL3.10(x)1.13i** | 0.226 +/- 9.60E-02 | 0.524 +/- 1.77E-02 | 0.29 +/- 0.081 | 0.666 +/- 0.128 |
| **DCL1.13(x)4.9i** | 0.527 +/- 0.144 | 0.667 +/- 0.281 | 1.158 +/- 0.281 | 0.465 +/- 0.086 |
| **DCL2.11(x)3.10i** | 0.965 +/- 4.94E-02 | 0.074 +/-5.99E-02 | 0.63 +/- 0.113 | 1.052 +/- 0.059 |
| **DCL4.9(x)3.10i** | 1.01 +/- 0.164 | 0.696 +/- 4.83E-0.2 | 0.228 +/- 2.85E-02 | 0.162 +/- 2.19E-02 |
| **DCL3.10(x)2/4.5i** | 0.733 +/- 0.657 | 0.007 +/-0.011 | 0.010 +/- 0.003 | 7.194E-14 +/- 3.484E-14 |
| **DCL2/4.16(x)1.13i** | 0.238 +/- 0.095 | 0.019 +/- 0.012 | 0.755 +/- 0.295 | 0.067 +/- 0.013 |

**Table S1:**  Transcript levels of DCLi single and crossed plant lines (adapted from Katsarou *et al.*, 2016)
