# Supplementary material for: DCL‐suppressed Nicotiana benthamiana plants: valuable tools in research and biotechnology
Source: Mol Plant Pathol. 2018 Dec 19;20(3):432–46. doi: 10.1111/mpp.12761 (PMC6637889; doi:10.1111/mpp.12761)
Supplement: Supplementary file 7 — Table S2 Small RNA deep sequencing libraries. [file MPP-20-432-s007.docx]

**Table S2:** Small RNAs deep sequencing libraries

| **Plant name** | **Library(reads)** |
| --- | --- |
| **WT** | 13521296 |
| **DCL1.13i** | 18399778 |
| **DCL2.11i** | 11446798 |
| **DCL3.10i** | 13246296 |
| **DCL4.9i** | 16136156 |
| **DCL1.13(x)2.11i** | 11976424 |
| **DCL2/4.5i** | 12820738 |
| **DCL3.10(x)2/4.5i** | 9867657 |
