# Supplementary material for: DCL‐suppressed Nicotiana benthamiana plants: valuable tools in research and biotechnology
Source: Mol Plant Pathol. 2018 Dec 19;20(3):432–46. doi: 10.1111/mpp.12761 (PMC6637889; doi:10.1111/mpp.12761)
Supplement: Supplementary file 10 — Table S5 Primers used in this study. [file MPP-20-432-s010.docx]

**Table S5**: Primers used in this study

| **Name** | **Sequence (5’-3’)** | **Purpose** | **Used (T^o^C)** | **Reference (If any)** |
| --- | --- | --- | --- | --- |
| **TRV-RNA2-For** | CTTCTCCTACGCAAGCGTCT | cloning | 53 ^o^C | This study |
| **TRV-RNA2-Rev** | AGCACTGTTAGCCCGTGAGT | cloning | 53 ^o^C | This study |
| **mGFP4-For** | TATGAGTAAAGGAGAAGAACTTTTC | probe | 45^o^C | This study |
| **mGFP4-Rev** | TTTATTTGTATAGTTCATCCATGCC | probe | 45^o^C | This study |
| **TuMV-For** | CAAGCAATCTTTGAGGATTATG | PCR | 49^o^C | (Sa et al. 2003) |
| **TuMV-Rev** | TATTTCCCATAAGCGAGAATAC | PCR | 49^o^C | (Sa et al. 2003) |
| **qPCR primers** | | | | |
| **U1 cDNA synt** | GTGCAGGGTCCGAGGTAGGGGCTGCGCGAACGCAGGCC | RT |  | This study |
| **U1 Fw qPCR** | CTGCCTAAGGTCGGCTCAAG | qPCR | 60^o^C | This study |
| **U4 cDNA synt** | GTGCAGGGTCCGAGGTAGGGCTTTACCCCATGAAGGG | RT |  | This study |
| **U4 Fw qPCR** | TTTCCAAACCCCCTCTTCGG | qPCR | 65^o^C | This study |
| **U6 cDNA synt** | GTGCAGGGTCCGAGGTTTTGGACCATTTCTCGAT | RT |  | (Turner et al. 2013) |
| **U6 Fw qPCR** | GGAACGATACAGAGAAGATTAGCA | qPCR | 60 ^o^C | (Turner et al. 2013) |
| **nbt-miR159** | TTTGGATTGAAGGGAGCTCTA | Reads : 804730 | | |
| **mir159 RT qPCR** | GTCGTATCCAGTGCAGGGTCCGAGGTATTCGCACTGGATACGACTAGAGC | RT |  | (Varkonyi-Gasic et al. 2007) |
| **mir159 Fw qPCR** | CGGCGGTTTGGATTGAAGGGA | qPCR | 65^o^C | (Varkonyi-Gasic et al. 2007) |
| **nbt-miR166b** | TCGGACCAGGCTTCATTCCTC | Reads : 154.462 | | |
| **mir166 RT qPCR** | GTCGTATCCAGTGCAGGGTCCGAGGTATTCGCACTGGATACGACGAGGAA | RT |  | This study |
| **mir166 Fw qPCR** | TCGCTTCGGACCAGGCTTCA | qPCR | 65^o^C | This study |
| **nbt-miR168d** | TCGCTTGGTGCAGGTCGGGAC | Reads : 32.083 | | |
| **mir168 RT qPCR** | GTCGTATCCAGTGCAGGGTCCGAGGTATTCGCACTGGATACGACGTCCCG | RT |  | This study |
| **mir168 Fw qPCR** | TGGTGTTCGCTTGGTGCAGGT | qPCR | 65^o^C | This study |
| **Nbe-miR396,3** | TTCCACAGCTTTCTTGAACTG | Reads : 359.849 | | |
| **mir396 RT qPCR** | GTCGTATCCAGTGCAGGGTCCGAGGTATTCGCACTGGATACGACCAGTTC | RT |  | This study |
| **mir396 Fw qPCR** | GCGTCGTTCCACAGCTTTCTT | qPCR | 65^o^C | This study |
| **Nb-miR397** | TCATTGAGTGCAGCGTTGATG | Reads : 13371 | | |
| **mir397 RT qPCR** | GTCGTATCCAGTGCAGGGTCCGAGGTATTCGCACTGGATACGACCATCAA | RT |  | This study |
| **mir397 Fw qPCR** | CGGCGTTCATTGAGTGCAGCG | qPCR | 65^o^C | This study |
| **Uni Rw miRNA qPCR** | GTGCAGGGTCCGAGGT | qPCR | 60-65^o^C | (Varkonyi-Gasic et al. 2007) |
| **qNbDCL2-F** | GAAGAACCACTTCTTAGGGGAAA | qPCR | 59^o^C | (Dadami et al. 2013) |
| **qNbDCL2-R** | GGCCATAACAAGGACTCAA | qPCR | 59^o^C | (Dadami et al. 2013) |
| **DCL4_For b** | GGGGAGGAATTGAATCTTCTG | qPCR | 60 ^o^C | (Kotakis et al. 2010) |
| **DCL4_Rev b** | TCCAGTATGAATGGCCACAA | qPCR | 60 ^o^C | (Kotakis et al. 2010) |
| **F-Box-F** | GGCACTCACAAACGTCTATTTC | qPCR | 62^o^C | (Liu et al. 2012) |
| **F-Box-R** | ACCTGGGAGGCATCCTGCTTAT | qPCR | 62^o^C | (Liu et al. 2012) |
| **L23-F** | AAGGATGCCGTGAAGAAGATGT | qPCR | 60^o^C | (Liu et al. 2012) |
| **L23-R** | GCATCGTAGTCAGGAGTCAACC | qPCR | 60^o^C | (Liu et al. 2012) |
| **PP2A-F** | GACCCTGATGTTGATGTTCGCT | qPCR | 60^o^C | (Liu et al. 2012) |
| **PP2A-R** | GAGGGATTTGAAGAGAGATTTC | qPCR | 60^o^C | (Liu et al. 2012) |

**Supplemental Bibliography For Table S5**

Dadami, E. et al., 2013. DICER-LIKE 4 but not DICER-LIKE 2 may have a positive effect on potato spindle tuber viroid accumulation in Nicotiana benthamiana. *Molecular plant*, 6(1), pp.232–234.

Kotakis, C. et al., 2010. Light intensity affects RNA silencing of a transgene in Nicotiana benthamiana plants. *BMC plant biology*, 10(1), p.220.

Liu, D. et al., 2012. Validation of Reference Genes for Gene Expression Studies in Virus-Infected Nicotiana benthamiana Using Quantitative Real-Time PCR. *PLoS ONE*, 7(9), p.e46451.

Sa, F. et al., 2003. Strains of Turnip mosaic potyvirus as defined by the molecular analysis of the coat protein gene of the virus. *Virus research*, 94, pp.33–43.

Turner, M., Adhikari, S. & Subramanian, S., 2013. Optimizing stem-loop qPCR assays through multiplexed cDNA synthesis of U6 and miRNAs. *Plant signaling and behavior*, 8(8), p.e24918. Available at: www.landesbioscience.com.

Varkonyi-Gasic, E. et al., 2007. Protocol : a highly sensitive RT-PCR method for detection and quantification of microRNAs. *Plant Methods*, 3(12), pp.1–12.
